# Identification of gut microbial species linked with disease variability in a widely used mouse model of colitis

**DOI:** 10.1038/s41564-022-01094-z

**Published:** 2022-04-01

**Authors:** Samuel C. Forster, Simon Clare, Benjamin S. Beresford-Jones, Katherine Harcourt, George Notley, Mark D. Stares, Nitin Kumar, Amelia T. Soderholm, Anne Adoum, Hannah Wong, Bélen Morón, Cordelia Brandt, Gordon Dougan, David J. Adams, Kevin J. Maloy, Virginia A. Pedicord, Trevor D. Lawley

**Affiliations:** 1 Experimental Cancer Genetics Lab, Wellcome Sanger Institute, Hinxton, UK; 2 Centre for Innate Immunity and Infectious Diseases, Hudson Institute of Medical Research, Clayton, Victoria Australia; 3 Department of Molecular and Translational Sciences, Monash University, Clayton, Victoria Australia; 4 Cambridge Institute of Therapeutic Immunology and Infectious Disease, Jeffrey Cheah Biomedical Centre, Cambridge, UK; 5 Department of Medicine, University of Cambridge School of Clinical Medicine, Cambridge, UK; 6 Animal Health Trust, Newmarket, UK; 7 Experimental Medicine Division, University of Oxford, Oxford, UK; 8 Institute of Infection, Immunity and Inflammation, University of Glasgow, Glasgow, UK

**Keywords:** Microbial communities, Inflammation, Gastrointestinal models

## Abstract

Experimental mouse models are central to basic biomedical research; however, variability exists across genetically identical mice and mouse facilities making comparisons difficult. Whether specific indigenous gut bacteria drive immunophenotypic variability in mouse models of human disease remains poorly understood. We performed a large-scale experiment using 579 genetically identical laboratory mice from a single animal facility, designed to identify the causes of disease variability in the widely used dextran sulphate sodium mouse model of inflammatory bowel disease. Commonly used treatment endpoint measures—weight loss and intestinal pathology—showed limited correlation and varied across mouse lineages. Analysis of the gut microbiome, coupled with machine learning and targeted anaerobic culturing, identified and isolated two previously undescribed species, *Duncaniella muricolitica* and *Alistipes okayasuensis*, and demonstrated that they exert dominant effects in the dextran sulphate sodium model leading to variable treatment endpoint measures. We show that the identified gut microbial species are common, but not ubiquitous, in mouse facilities around the world, and suggest that researchers monitor for these species to provide experimental design opportunities for improved mouse models of human intestinal diseases.

## Abstract (web summary)

Large-scale gut microbiome analysis of a widely use mouse model of inflammatory bowel disease reveals that the gut microbiome is a driver of variability across genetically identical mice, in particular two species that are associated with variable treatment endpoints.

## Main

Experimental mouse models are central to basic biomedical research and serve as important preclinical models in drug discovery for a variety of human diseases, including autoimmune and metabolic disorders, cancers and infections. Unfortunately, there is tremendous variability in disease penetrance and reproducibility among genetically identical mice within and between mouse facilities, limiting the utility of many commonly used models^1–3^. Specific-pathogen-free (SPF) protocols have been successfully employed to control the confounding effects that can be caused by known bacterial pathogens, but we lack equivalent knowledge of the effects of the diverse, uncharacterized symbiotic bacteria within the indigenous gut microbiota of mice. While our knowledge of the human gut microbiota in health and disease has expanded extensively with large-scale microbiome studies, comprehensive computational bacterial discovery^4,5^ and extensive genome-sequenced bacterial collections^6–9^, equivalent investigations in mouse models have remained limited. The vast differences in species type, composition and dispersal between human and mouse microbiotas highlight the importance of efforts to culture mouse-specific bacteria and to validate their functions during intestinal homeostasis and disease^10,11^.

Inflammatory bowel disease (IBD) research relies heavily on laboratory mice to understand colitis and the role of host genetics in disease resistance and susceptibility. Detailed immune characterization shows colitis induction with specific microbial communities^12^ or species colonization with *Helicobacter* spp.^13,14^, Enterobacteriaceae^15^, *Bilophila wadsworthia*^16^ or segmented filamentous bacteria^17^. Potential genetic modulators of IBD are routinely assessed through administration of dextran sulphate sodium (DSS) at various concentrations in drinking water, which acts by damaging the gut epithelial barrier to trigger inflammatory disease^12,13^. In germ-free mice, immune-mediated colitis is largely absent although reduced barrier function can introduce susceptibility at higher DSS concentrations^18^. Similarly, dietary changes in both germ-free and SPF mice can determine DSS susceptibility in a microbiome-dependent and -independent manner^19^, and highly specific protection is provided by the presence of *Clostridium immunis*^20–23^. Despite these advances, the gut microbiota in these mouse models, including standard SPF models, is rarely profiled at the genomic level or cultured in the laboratory, nor robustly controlled to understand the contribution of general gut bacteria to experimental outcomes.

To quantify the variability of the widely used DSS model of IBD and to investigate primary factors contributing to this variability, we undertook a large-scale experiment using 579 genetically identical laboratory mice in a single animal facility. Incorporating standardized phenotyping, microbiome analysis and metadata collection, this approach was designed to identify and validate the causes of variability for standard experimental endpoints (weight loss and intestinal pathology). Using a data-driven approach we identified, from gut microbiome data, candidate bacterial taxa driving weight loss and intestinal inflammation. Functional validation with cultured isolates suggests that these specific bacterial strains can determine experimental outcomes in gnotobiotic mice. Thus, using an experimental approach for microbiome discovery, we identified the previously undescribed mouse gut bacteria *Duncaniella muricolitica* and *Alistipes okayasuensis*, found frequently in mouse colonies around the world, that impact outcomes in the DSS model of colitis and may be acting as pathobionts.

## Results

### Disease severity is highly variable in the DSS mouse model

**Fig. 1:**
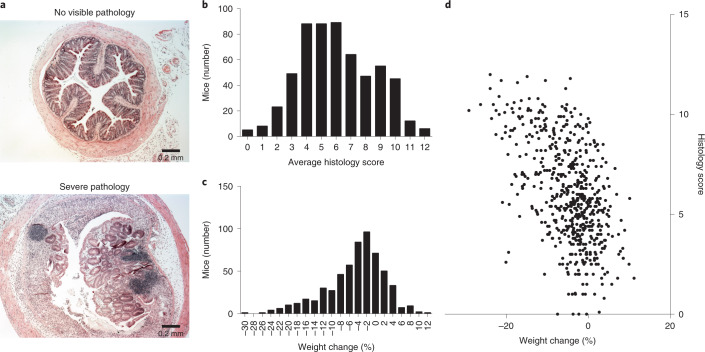
Disease severity is highly variable in the DSS mouse model. **a**, Representative images of extremes of midcolon inflammatory response in wild-type C57/BL6 mice after treatment with DSS, showing a score of 0 (top) and a score of 12 (bottom). **b**,**c**, Distribution of average histology scores (**b**) and weight change (**c**) across 579 wild-type C57BL/6 N mice (297 males, 282 females; median age 11 weeks; s.d. 12 days) exposed to DSS treatment. **d**, Relationship between weight change and histology scores across all 579 mice. *R*^2^ = 0.273.

We administered 1.5% DSS through the drinking water for 7 days to 579 identically housed, SPF wild-type C57BL/6N mice (297 males, 282 females; median age 11 weeks; s.d. 12 days) from 14 distinct parental lineages (that is, founder matings derived from a single breeding pair). We observed extensive variability in experimental endpoints in these genetically identical mice at 10 days post DSS treatment, with histological assessment of intestinal pathology of the mid and distal colon ranging from severe inflammation, characterized by extensive crypt loss, leukocytic infiltration and edema, to no visible inflammation (Fig. 1a,b). Substantial variation was also observed in the weight change of mice treated with DSS, with the response ranging from 12% weight gain to 30% weight loss across the cohort (Fig. 1c). Regression analysis considering histology scores and weight loss demonstrated limited correlation (*R*^2^ = 0.27), suggesting that weight loss and intestinal pathology outcomes, common experimental endpoint measures in the DSS model, may be partially independent responses to DSS (Fig. 1d). This relationship is consistent with systemic complications contributing to experimental endpoints in the DSS model.

### Gut microbiota is major driver of variable response to DSS

**Extended Data Fig. 1:**
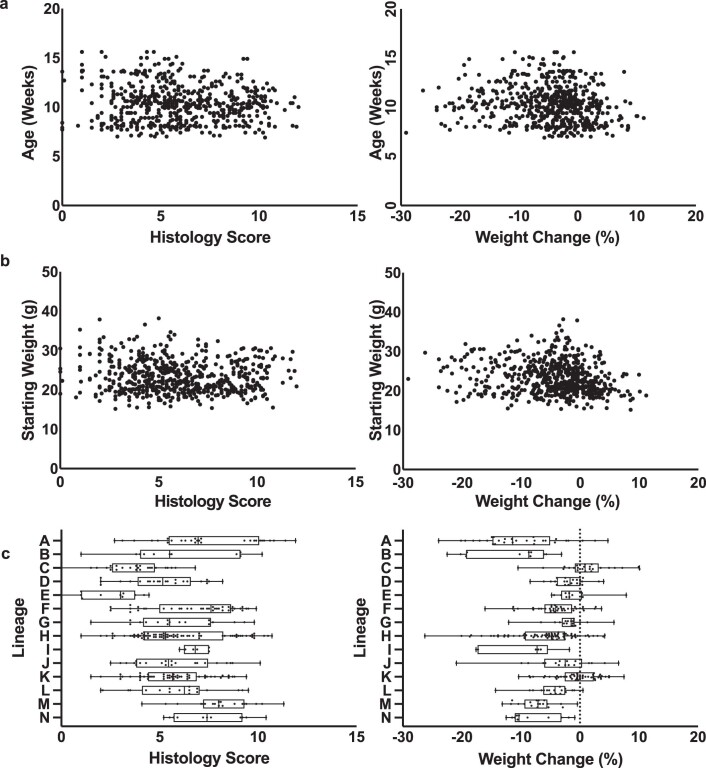
Lineage specific variation across C57BL/6 mice.

To identify the primary factors determining disease outcome, we established a random forest classifier model incorporating mouse sex, age, lineage, parents, mouse room and experimental date to assess the relative importance of each of these factors (Extended Data Fig. 1). The strongest predictive factor for weight loss and histology score, both together and independently in these genetically identical C57BL/6N mice maintained under standardized SPF conditions within the same facility, was parental lineage, with correlation analysis confirming these associations (*P* < 0.01, Kruskal–Wallis; Extended Data Fig. 1).

**Fig. 2:**
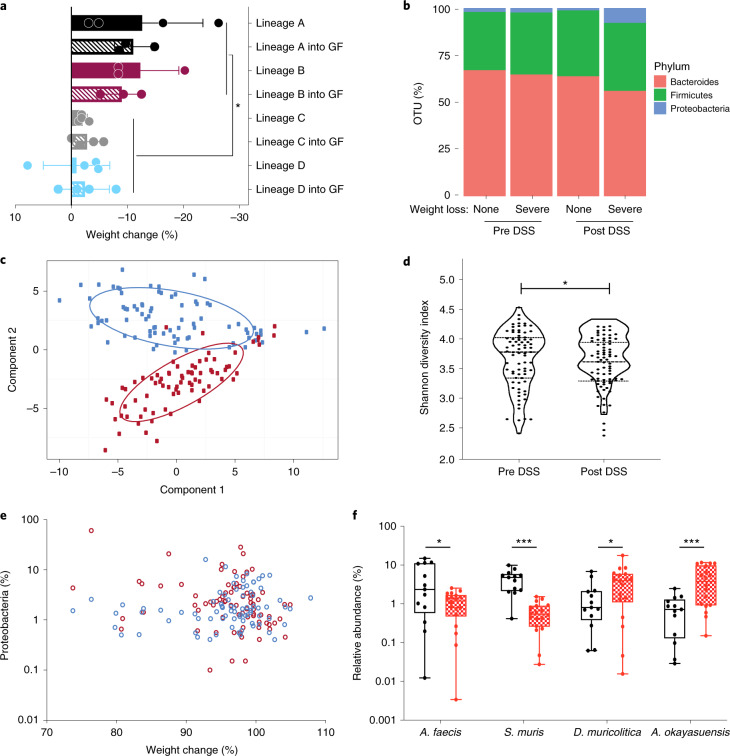
Discovery of candidate bacterial taxa associated with DSS disease variability. **a**, Comparison of weight change observed in conventional SPF mice (lineages A, B, C and D, solid colors) and germ-free (GF) mice colonized by oral gavage (lineages A, B, C and D into GF, hatched colors) after 10-day DSS challenge (*P* = 0.0002, two-tailed *t*-test with Welch’s correction; data plotted as mean ± s.d.; *n* = 4 (lineages A, C, C + GF, D and D + GF) and *n* = 3 (lineages B, B + GF, A + GF)). **b**, Distribution of Bacteroides (red), Firmicutes (green) and Proteobacteria (blue) in pre- and post-DSS samples exhibiting asymptomatic or >5% weight loss and inflammation. **c**, Principal component analysis of 16 S rRNA amplicon sequencing profiling identifies a clear difference between pre-DSS samples (blue) and post-DSS samples (red), regardless of lineage. **d**, Alpha diversity using Shannon diversity index grouped by pre- and post-DSS samples (*P = 0.0357, paired *t-*test; *n* = 77). **e**, Relationship between weight change and relative abundance of Proteobacteria pre DSS challenge (blue; Spearman correlation, 0.196) and post DSS (red; Spearman correlation, −0.289). **f**, Relative abundance of *A. faecis*, *A. okayasuensis*, *D. muricolitica* and *S. muris* before challenge with DSS amongst samples from mice that experienced >5% weight loss (red) or no weight loss (black) (*P* = 0.0264, 0.0006, 0.0351, 0.0010, two-tailed *t*-test; *n* = 18 and *n* = 13 independent animals, respectively. Minima (25th percentile) median (75th percentile) and maxima are shown. *P < 0.05; ***P ≤ 0.001). Source data

Since all mice were derived from the same genetic founder stock, the most likely mediator of their DSS phenotype was an extrinsic lineage-specific factor. However, to exclude the role of host genetics or epigenetic differences between colonies, and to determine whether the microbiota alone could be responsible for the observed phenotypic variation, fecal samples were collected from SPF mice (without DSS treatment) from parental lineages that represented extremes of disease outcome and were used to colonize C57BL/6N germ-free mice. These colonized recipient mice were then subjected to DSS treatment involving daily weight measurements, with colon sections collected and analyzed histologically 10 days after the initiation of DSS treatment. Recipient mice exhibited both weight loss and pathology scores not statistically different from those observed in the respective donor mouse lineages, but differing significantly between disease- and non-associated lineages (*P* = 0.0002, paired *t*-test; Fig. 2a). From these results it is clear that, independent of other factors such as host genetics or epigenetic lineage-specific factors, the microbiota is sufficient to produce the observed variation in phenotypic response to DSS administration.

### Discovery of bacteria that drive disease variability

**Extended Data Fig. 2:**
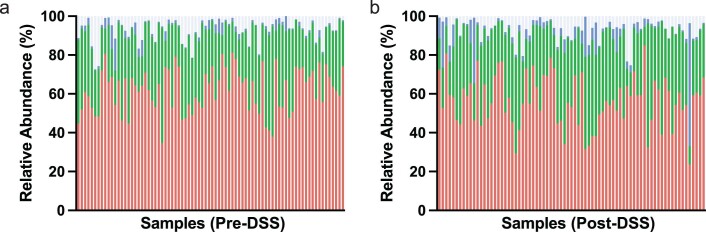
Phyla level microbiome composition Pre- and Post- DSS.

**Extended Data Fig. 3:**
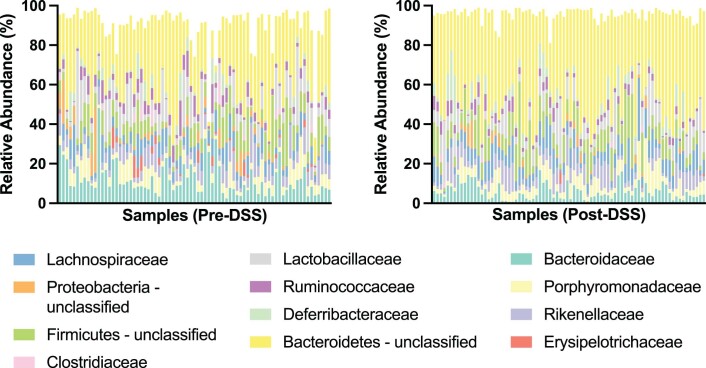
Family level microbiome composition Pre- and Post- DSS.

Having established the importance of the microbiota in mediation of phenotypic outcomes to DSS in genetically identical mice, we next sought to understand the degree of variability in the mouse microbiota across a single facility, and to define changes in the microbiota that occur with DSS treatment. Fecal samples were collected before DSS treatment and 10 days after DSS exposure, and subjected to taxonomic profiling with 16 S ribosomal RNA amplicon sequencing to determine microbiota community composition. Consistent with previous reports of gastrointestinal inflammation, this analysis demonstrated a clear separation of microbiome community between pre- and post-DSS samples (Fig. 2b,c and Extended Data Figs. 2 and 3) and a significant reduction in bacterial diversity (Shannon diversity index, *P* = 0.0357; Fig. 2d)^12^. Although expansion of Proteobacteria is often associated with gut microbiome dysbiosis, while an increase in Proteobacteria was observed post DSS (*P* < 0.05, Kolmogorov–Smirnov test), limited association was observed between weight loss and Proteobacteria proportion either pre (Fig. 2e; Spearman correlation, 0.196) or post DSS treatment (Fig. 2e; Spearman correlation, −0.289). This suggests that other gut bacteria may be contributing to the observed variation in DSS resistance and susceptibility.

Given the transmissibility of disease phenotype we observed in the germ-free mouse experiments, we reasoned that the observed phenotypic variation was primarily dependent on baseline microbiota composition before DSS treatment. To understand the composition of microbiota associated with increased risk of disease outcome, we applied linear discriminant analysis to gut microbiota composition before and after DSS exposure^24^. This unsupervised analysis leveraged the variability between mouse lineage-specific bacteria to identify the bacterial taxa that most closely predict DSS response. Using this approach, we identified two statistically different bacterial taxa associated with no disease, and five statistically different bacterial taxa associated with >5% weight loss. Notably, no relationship was observed between the occurrence or abundance of these isolates in mice before DSS exposure, and equivalent predictive bacterial taxa were not identified within the microbiota of mice after DSS exposure. Our results suggest that the presence of specific gut bacterial taxa before DSS exposure may influence disease severity and outcome, possibly by either priming or protection from disease.

### Isolation of bacterial species for functional validation

**Extended Data Fig. 4:**
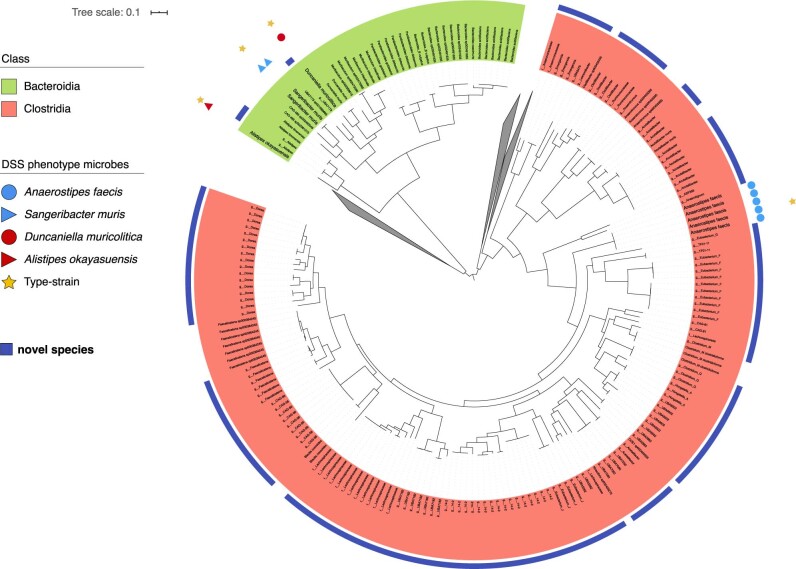
Genomic Characterisation of Bacterial Isolates.

While we show clear associations between specific bacterial taxa and DSS-mediated disease outcome using 16 S ribosomal RNA gene sequencing, accurate taxonomic identification to strain level and experimental validation require the isolation and genome sequencing of pure clonal cultures of bacterial strains. To target and purify bacterial strains, we next performed broad-based culture of ~2,000 bacterial colonies from mouse fecal samples. The resulting isolates were identified by capillary 16 S rRNA gene sequencing and included four previously identified bacterial taxa, two ‘disease associated’ and two ‘health associated’. These isolates were subjected to whole-genome sequencing and genomic analysis to aid in precise taxonomic and phylogenetic assignment (Extended Data Fig. 4). In addition, we performed extensive phenotypic characterization and comparative genome analyses of these four species (Methods) as a basis to provisionally name the health-associated bacteria *Anaerostipes faecis* and *Sangeribacter muris* and the disease-associated bacteria *Duncaniella muricolitica* and *Alistipes okayasuensis*; of note, the last three species are mouse specific. Examination of the relative abundance of each of these species before DSS treatment identified significantly higher levels of *A. faecis* (*P* = 0264, *t*-test) and *S. muris* (*P* = 0.0010, *t*-test) in those mice without weight loss, and significantly higher levels of *D. muricolitica* (*P* = 0.0351, *t*-test) and *A. okayasuensis* (*P* = 0.0006, *t*-test) in mice that experienced >5% weight loss (Fig. 2f).

**Fig. 3:**
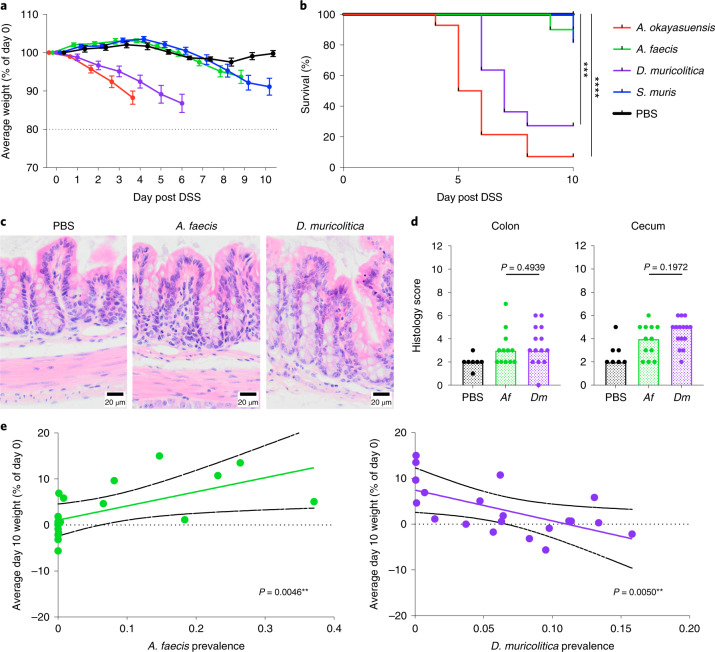
Phenotypic analysis of mice monocolonized with candidate bacterial taxa. **a**,**b**, Average weight loss/gain (**a**) and survival (**b**) in gnotobiotic mice precolonized with either *A. okayasuensis* (red; *n* = 14 animals, *P* = 0.00001), *A. faecis* (green; *n* = 10 animals, *P* = 0.31731), *D. muricolitica* (purple; *n* = 10 animals, *P* = 0.00074) or *S. muris* (blue; *n* = 11 animals, *P* = 0.16653) and in germ-free controls (PBS; black; *n* = 10 animals) in response to oral DSS challenge. All survival curves were compared to PBS control by log-rank (Mantel–Cox) test, with median and interquartile range plotted. **c**,**d**, Representative colon images (400×) from two experiments, each with between three and seven mice per group (**c**), and histological scores from H&E staining of midcolon (**d**) (*P* = 0.4939, Mann–Whitney test) and cecum (*P* = 0.1972, Mann–Whitney test) from either control germ-free mice (PBS) or mice monocolonized with either *A. faecis* (*Af*, *n* = 12) or *D. muricolitica* (*Df*, *n* = 14) and challenged with oral DSS for 7 days. Average weight loss curves were censored after the first mouse in each group reached 80% of baseline weight. **e**, Day 10 weight loss relative to day 0 starting weight in 20 mice, showing a positive correlation (green line) between weight change and prevalence in *A. faecis* (*R*^2^ = 0.3679, *P* = 0.0046) and negative correlation (purple line) between weight change and prevalence in *D. muricolitica* (*R*^2^ = 0.3626, *P* = 0.005). Solid black lines indicate 95% confidence interval. *P ≤ 0.05, **P ≤ 0.01, ***P ≤ 0.001, ****P ≤ 0.0001. Source data

**Extended Data Fig. 5:**
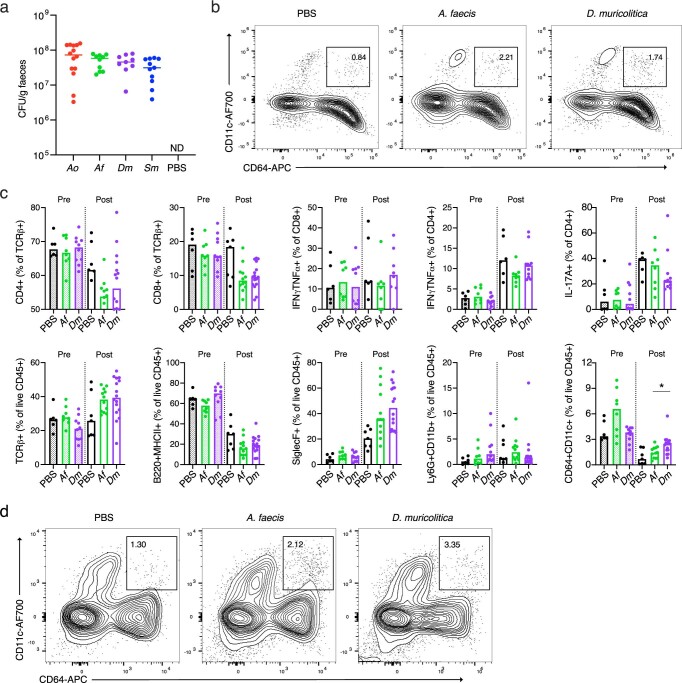
Mono-colonisation of germ-free mice.

**Extended Data Fig. 6:**
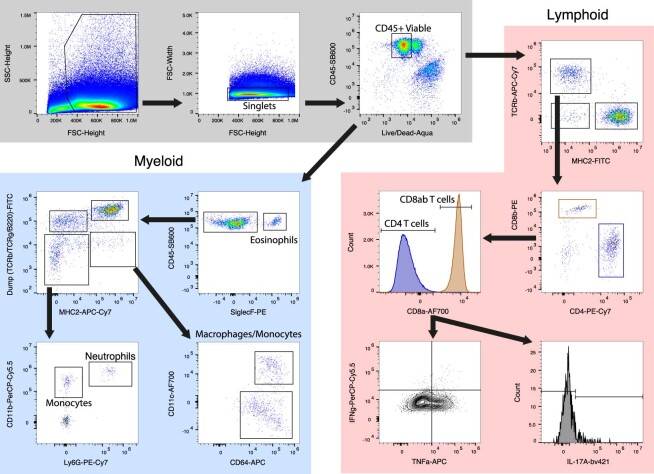
Flow cytometric gating strategy.

We next repeated the DSS experimental model in either germ-free mice or germ-free mice stably monocolonized for 4 weeks with one of these four bacterial strains (Extended Data Figs. 5a and 6). Control germ-free mice treated with DSS neither lost weight (Fig. 3a) nor experienced mortality (Fig. 3b) over the course of the experiment, suggesting that the presence of gut bacteria is important in triggering overt disease in our facility. Comparable experimental outcomes were observed in DSS-treated, germ-free mice monocolonized with either of the health-associated bacteria, which did not lose weight or develop intestinal pathology (Fig. 3a) nor experienced significant mortality (Fig. 3b). In contrast, when DSS-treated mice were monocolonized with either of the disease-associated bacteria, we observed more rapid and greater weight loss (Fig. 3a) and significantly reduced survival (Fig. 3b). Therefore, we identified and validated *D. muricolitica* and *A. okayasuensis* as mouse bacteria that can promote disease not observed in mice monocolonized with the commensal bacteria *S. muris* and *A. faecis*.

To understand inflammatory immune responses in the intestine potentially caused by disease-associated versus health-associated species, we next examined histology, immune cell populations and cytokine production. The human-colonizing *A. faecis* and the facility-prevalent *D. muricolitica* were chosen as exemplary disease- and health-associated bacteria, respectively, for these analyses. Flow cytometric analysis of intestinal lamina propria leukocytes isolated from germ-free mice monocolonized for 4 weeks with either *A. faecis* or *D. muricolitica* revealed that colonization with either strain did not significantly impact the composition of intestinal leukocyte and lymphocyte subsets (Extended Data Figs. 5b,c and 6). However, the perturbation and epithelial disruption induced by DSS led to significantly increased inflammatory CD64^+^CD11c^+^ monocytes/macrophages in the lamina propria compartment of the large intestine in *D. muricolitica* monocolonized mice, but not in *A. faecis* monocolonized mice (Extended Data Figs. 5c,d and 6). These monocytes have been shown to play a pathogenic role in the intestine in the context of *Helicobacter*-induced inflammation^25^. This suggests that, although neither pathogenic nor immunogenic at homeostasis, *D. muricolitica* drives increased inflammatory responses in the wake of epithelial damage whereas health-associated *A. faecis* does not. Importantly, while histological samples taken after 7 days of DSS treatment from colons and caeca of *D. muricolitica* monocolonized mice occasionally exhibited features of severe inflammation, this was not statistically different from that of *A. faecis* monocolonized mice (Fig. 3c,d) and did not consistently correspond to the weight loss observed. This again suggests that weight loss in the DSS model may not always correlate with intestinal inflammation and pathology.

To determine whether the presence of *D. muricolitica* or *A. faecis* had a positive or negative correlation with weight change, we next examined 20 independent mice and assessed the relationship between bacterial levels using quantitative PCR (qPCR) before DSS treatment and weight change observed at day 10. This analysis identified a positive correlation between weight change and pre-DSS *A. faecis* abundance (*R* = 0.3679, *P* = 0.0046; Fig. 3e). Similarly, a negative correlation was observed between weight change and *D. muricolitica* abundance (*R*^2^ = 0.3626, *P* = 0.0050; Fig. 3e). This independent confirmation provides further validation of the relationship between these species and the severity of DSS colitis; however, additional work is needed to confirm their roles as pathobionts.

### Global distribution and prevalence in other mouse facilities

Because *D. muricolitica* and *A. okayasuensis* affected the outcome of DSS colitis in our mouse facility, we next explored the presence and distribution of these bacteria in other mouse facilities around the world. To do so, we first generated a global representation of laboratory mouse intestinal microbiomes by curating 582 publicly available shotgun metagenome samples from ‘control’, SPF mice. We combined this dataset with shotgun metagenomes from feces of 40 mice at the Wellcome Sanger Institute to yield a global mouse gut microbiome dataset covering 31 institutes across 12 countries. We then determined the prevalence and abundance of health-associated bacteria *S. muris* and *A. faecis* and disease-associated bacteria *D. muricolitica* and *A. okayasuensis* in this mouse microbiome dataset.

**Fig. 4:**
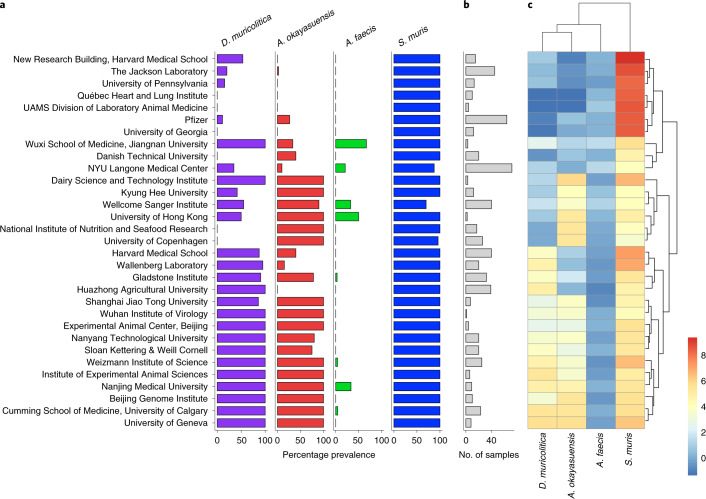
Global epidemiology and intrainstitutional variation in disease- and health-associated species. **a**–**c**, Prevalence and abundance of disease- and health-associated species in SPF mice across international mouse facilities. **a**, Intrainstitute prevalence of *D. muricolitica* (purple), *A. okayasuensis* (red), *A. faecis* (green) and *S. muris* (blue). A species is defined as present in a sample if ≥0.01% shotgun metagenomic reads are assigned to it. Intrainstitute prevalence is calculated as the percentage of samples per institute in which a species is present. **b**, Bar plot indicating number of samples per institute. **c**, Heatmap of intrainstitute mean abundance for each species. In **b** and **c**, Institute names correspond with those in **a**. For mean abundance, data are the percentage of classified reads assigned to each species, with zero counts removed using Bayesian multiplicative replacement, followed by center log-ratio transformation.

*Duncaniella muricolitica* and *A. okayasuensis* were both dominant members of the mouse microbiome, present in 54.5 and 47.9% of samples and with a mean abundance of 0.52 and 0.48% reads per sample, respectively. Importantly, *D. muricolitica* and *A. okayasuensis* were each detected in 80.6% (25/31) of the institutes included in the analyses (Fig. 4), and there were only three institutes that did not contain either of these species. For comparison, among health-associated species *S. muris* was the most dominant in the mouse gut microbiota, present in 96.5% of samples with a mean abundance of 6.2% reads, while *A. faecis* was a relatively minor species, present in 5.9% of samples with a mean abundance of 0.22% reads. *S. muris* was present in all (31/31) of the institutes included in the analyses, whereas *A. faecis* was detected in 25.8% (8/31). The abundance of each species also differed between institutes (Fig. 4). Thus, we show that mouse-derived bacteria cultured from our institute are common mouse symbionts and, importantly, *D. muricolitica* and *A. okayasuensis* are common, but not ubiquitous, in animal facilities around the world.

## Discussion

Despite experimental variability in the DSS mouse model, which limits the interpretation and translation of data, the model is increasingly used in basic research (>6,000 DSS model publications on PubMed) and by the pharmaceutical and biotechnology industries. Cohousing, heterozygote mating and other experimental procedures have been used in an attempt to normalize the microbiota within experiments and ensure direct measures of the effects of mouse genetic background on disease phenotype rather than indirect and unknown microbiota-associated effects^26^. In our approach we leveraged this variability in DSS outcome, in combination with microbiome analysis, machine learning, anerobic culturing and germ-free mouse validation experiments, to gain relevant biological insight into microbiota determinants of DSS disease. While this consideration of the microbiome did not completely eliminate the impact of multidimensionality in the DSS model, we identified and undertook experimental validation of *D. muricolitica* and *A. okayasuensis*, both of which have a dominant effect on DSS outcome.

Interestingly, *D. muricolitica* and *A. okayasuensis* are phylogenetically distinct from each other and neither had computationally predicted virulence factors within their genome. While it remains unclear how these isolates contribute to DSS disease, both exhibit a potential for polysaccharide utilization that may mediate this relationship through interaction with the mucus layer. We also noted that weight loss and intestinal pathology do not always correlate, so they should not necessarily be used as surrogates for each other. Similarly, substantial weight loss was observed in monocolonized mice even in the absence of severe inflammation, suggesting that activation of key pathways alone can have a substantial impact on experimental outcome. Together, these insights raise the need for further experimental study of host interactions and the biology of these important mouse-specific bacteria.

We propose that researchers should consider monitoring for *D. muricolitica* and *A. okayasuensis* in their mouse facilities, as is undertaken for other immunophenotyped-affecting microbes such as segmented filamentous bacteria^27^. In some cases, precolonization of animals with these bacteria, if they are not present in the facility, may yield better experimental control and increase confidence in this important and widely used mouse model of human disease. Overall, our work suggests that the application of metagenomics techniques to report microbiota composition, in addition to the genetic and disease phenotypes being described, should represent a standard minimum requirement for the DSS mouse model. We have deposited the *D. muricolitis*, *A. okayasuensis*, *S. muris* and *A. faecis* strains and genomes in public repositories to support this work.

## Methods

### Mouse models

Mice were maintained under either germ-free or SPF conditions at the Wellcome Sanger Institute Home Office-approved facility, with all procedures carried out in accordance with the United Kingdom Animals (Scientific Procedures) Act of 1986 under Home Office approval (PPL no. 80/2643). Germ-free mice were maintained in positive-pressure isolators (Bell), with feces tested by culture, microscopy and PCR to ensure sterility. Consumables were autoclaved at 121 °C for 15 min before introduction into the isolators. For experimentation, cages were opened in a vaporized hydrogen peroxide-sterilized, class II cabinet (Bioquell), with fecal transplant and monocolonized gnotobiotic lines generated by weekly oral gavage over a 3-week period. Materials were prepared in Dulbecco’s PBS at 100 mg ml^–1^ immediately before administration under anaerobic conditions (10% H, 10% CO_2_, 80% N) in a Whitley DG250 workstation at 37 °C (ref. ^8^). Mice were maintained in sterile ISOcages (Tecniplast) and housed on ISOrack for the period of the experiment.

### DSS colitis challenge

Dextran sulphate sodium was administered at a concentration of 1.5% (w/v) (Affymetrix, Inc., average molecular weight 44 kDa) in drinking water for 7 days, followed by 3 days with regular drinking water, to C57BL6/6N mice aged 7–16 weeks (297 male, 282 female; median age 11 weeks; s.d. 12 days). Mice were weighed every day and culled if weight loss reached 20% of starting weight. Average weight loss curves were censored for each group when the first member of the group reached this endpoint, to avoid misleading calculations of the average using the remaining members. No statistical methods were used to predetermine sample sizes in these experiments.

### Histological assessment of intestinal inflammation

Dextran sulphate sodium-treated SPF mice were sacrificed at day 10 by cervical dislocation, and samples from mid and distal colon taken. Tissue sections were fixed in buffered 10% formalin, paraffin embedded, cut and stained with hematoxylin and eosin (H&E). Colon histopathology was blind-graded semiquantitatively, on a scale from zero to three, for four criteria: (1) degree of epithelial hyperplasia/damage and goblet cell depletion; (2) leukocyte infiltration in lamina propria; (3) area of tissue affected; and (4) presence of markers of severe inflammation, including crypt abscesses, submucosal inflammation and edema. Scores for individual criteria were added for an overall inflammation score of between zero and 12 for each sample. Scores from mid and distal colon were then averaged to obtain inflammation scores for each mouse colon.

Monocolonized, germ-free mice were euthanized 7 days after DSS administration and samples taken from the cecum and midcolon. Tissue sections were fixed in methacarn, paraffin embedded, sectioned and stained with H&E. Colon and cecum histopathology was graded on a semiquantitative scale by a pathologist blinded to the groups. The criteria scored were (1) degree of epithelial hyperplasia/damage and goblet cell depletion; (2) severity of leukocyte infiltration in lamina propria; (3) extent of inflammation; and (4) presence of markers of severe inflammation, including crypt abscesses, submucosal inflammation and edema. Each criterion was scored on a scale of zero to three based on previously described thresholds^28^ and the sum of the individual scores was recorded as an indication of overall inflammation. For all animal experiments, treatments were randomized by cage by researchers blinded to treatment conditions. For statistical analysis, distribution was assumed to be normal but this was not formally tested

### Bacterial culture

Fecal samples and bacterial isolates were cultured for anaerobic gastrointestinal bacteria under anaerobic conditions (10% H, 10% CO_2_, 80% N) in a Whitley DG250 workstation at 37 °C (ref. ^8^). Fecal samples were homogenized in reduced PBS (0.1 g ml^–1^ PBS), serially diluted and plated directly onto YCFA agar supplemented with 0.002 g ml^−1^ each of glucose, maltose and cellobiose. Colonies were picked, restreaked to purity and identified using 16 S rRNA gene sequencing.

### Microbiota profiling and analysis

Fecal samples were collected directly from mice and immediately stored at −80 °C until DNA extraction. DNA was extracted from samples using the FastDNA Spin Kit for Soil (MPBio) and stored at −20 °C until metagenomic sequencing. DNA samples were quantified using a Qubit 4 Fluorometer (Thermo Fisher). 16 S rRNA amplicon-based profiling was performed using the FastDNA Spin Kit for Soil (MP Biomedicals) on 300 mg of fecal sample from each mouse. The V1–V2 region of 16 S rRNA genes was amplified with a Q5 High-Fidelity Polymerase Kit (New England Biolabs): F': AATGATACGGCGACCACCGAGATCTACAC-TATGGTAATT-CC-AGMGTTYGATYMTGGCTCAG; R': CAAGCAGAAGACGGCATACGAGATACGAGACTGATTAGTCAGTCAGAAGCTGCCTCCCGTAGGAG. Each PCR amplification was performed as four independent amplifications and pooled in equimolar amounts for 150-base-paired (bp)-end sequencing with the Illumina MiSeq platform. For shotgun metagenomic sequencing, samples with >100 ng of DNA material were processed onward to paired-end (2 × 150 bp) metagenomics sequencing on the HiSeq 4000 platform as per standard manufacturer library preparation and sequencing protocols.

### Metagenomic data analysis

Analysis of 16 S rRNA amplicon sequences was performed with mothur MiSeq SOP v.1.42.3 using SILVA v.132 (ref. ^29^). The 16 S rRNA gene alignments were used to determine maximum likelihood phylogeny using FastTree v.2.1.10 (ref. ^30^). Phylogenetic trees were visualized and edited using iTOL^31^. Shotgun metagenomic reads were filtered for quality and adapter sequences using KneadData v.0.7.3, with default settings. Host reads were removed from samples using the GRCm39 reference genome and Bowtie2 v.2.3.5. In addition, reads were aligned to the Phi X 174 genome and removed. Taxonomic classification of metagenomic reads was performed using Kraken2 v.2.0.8 and a custom database built from the genomes of the Mouse Gut Bacterial Catalog (MGBC).

All data analyses were performed in R v.4.0.2. Species abundant at ≥0.01% reads were considered present in a sample, and operational taxonomic units (OTUs) were considered statistically different at *P* < 0.05. For shotgun metagenomic sequencing, relative abundance of species was corrected using Bracken v.2.5.2. Bar plots for species prevalence data and sample numbers were generated using the ggplot2 v.3.3.3 package. To quantify and visualize intrainstitute abundance, species abundance data for each sample underwent Bayesian multiplicative replacement of zero counts using the zCompositions v.1.3.4 package, and the data transformed using center log-ratio normalization. Mean abundance per sample was then calculated and visualized using the pheatmap v.1.0.12 package. Principal component analyses were performed in R, and linear regression analysis was performed using the linear regression function in python scikit-learn v.0.20.4. Alpha diversity was assessed using Vegan v.2.5.6 (ref. ^30^). Correlation analysis was performed using Spearman’s rank correlation coefficient.

### Curation of public metagenome samples

To assess the abundance of both health- and disease-associated bacteria in mice from different institutes, we curated publicly available fecal shotgun metagenomes for healthy, control SPF mice from NCBI and ENA. These samples included mice from different genetic backgrounds and strains, but samples were excluded if mice were younger than 3 weeks, had received antimicrobial treatment or dietary intervention or had gene knockout. In total 582 fecal shotgun metagenomes were curated.

### qPCR of bacterial load

*Anaerostipes faecis* and *D. muricolitica* were quantified in the feces of SPF mice using qPCR with custom primers. Primers were designed using the taxon-linked gene catalog of MGBC^32^. Briefly, we identified gene targets that were both unique to, and highly conserved in, the genome of each species. To ensure target specificity, we selected gene clusters that shared <50% sequence identity with all nontarget species sequences. Gene targets were defined as highly conserved if they were identified in 100% of high-quality target species genomes in the MGBC. Furthermore, only single-copy-number gene targets were considered. Sequences for selected gene products were then clustered at 90% sequence identity and used as templates for design of PCR primers with NCBI Primer-BLAST^33^, ensuring specificity against the NCBI nonredundant gene catalog. Two primers for each species were used in conjunction for qPCR.

Primers: A14_1 (gene target: MGBC000029_00009), forward 5'-AGCTGTTCAGGTGTCTGATCTT-3', reverse 5'-CGGTCCACTTCGCAGTATCA-3', product size 74 bp; A14_2 (gene target: MGBC000029_00377), forward 5'-ATGCAGCACTGGGAGATTCA-3', reverse 5'-CTGCGTGTCCACAGAAGAGT-3', product size 70 bp; A60_1 (gene target: MGBC109741_00862), forward 5'-CCATCTTTTCGGACGGTGGA-3', reverse 5'-CGAGGTCTCCCTGAAACGAC-3', product size 125 bp; A60_2 (gene target: MGBC140600_00802), forward 5'- AAAACGGTGACAGCTCGGAA-3', reverse 5'- GTCTCTTTTTGGCGGATGGG-3', product size 93 bp.

DNA was extracted from feces using FastDNA Spin Kit for Soil (MPBio) according to the manufacturer’s instructions, and DNA eluted into 100 µl of double-distilled H_2_O. Eluted DNA was then diluted 1:50 and qPCR performed using SYBR Green chemistry (Thermo Fisher). To approximate the relative abundance of target species in SPF feces, cycle threshold values were normalized to those of a universal bacterial 16 S primer (F: 5'- GTGSTGCAYGGYTGTCGTCA-3'; R: 5'- ACGTCRTCCMCACCTTCCTC-3').

### Intestinal immune cell isolation

Large intestines were excised and separated from mesentery and fat. Transluminal sections were taken from the cecum and distal colon for histology. The intestines were opened longitudinally, cleared of feces then washed three times in cold PBS. To isolate intraepithelial immune populations, the large intestines were cut into 1-cm pieces, washed in cold PBS then incubated in 10 ml of PBS + 1 mM dithiothreitol for 10 min. Tissues were manually disrupted via shaking and then strained, with supernatant then collected. Tissues were incubated in 10 ml of PBS + 30 mM EDTA + 10 ml of HEPES at 37 °C and 200 r.p.m., before being shaken and strained. The supernatants from these two steps were pooled, filtered at 70 µm and the filtrate fractionated using a discontinuous Percoll gradient (80/40%). Epithelial cells were isolated from the surface of the Percoll, and intraepithelial immune cells isolated from the interface.

To access the lamina propria compartment, tissues were manually chopped and digested in HBSS + 25 mM HEPES + 1 mM sodium pyruvate containing 0.05 mg ml^–1^ Collagenase VIII (Sigma) and 50 µg ml^–1^ DNase I (Sigma) for 1 h at 37 °C and 80 r.p.m. Samples were mechanically disrupted and filtered at 70 µm. The filtrate was fractionated using a discontinuous Percoll gradient (80/40%). Lamina propria immune cells were isolated from the interface.

### Flow cytometry

Intestinal and mesenteric lymph node immune cell populations were characterized by flow cytometry. For intracellular cytokine staining, cells were incubated in T cell medium (RPMI, 10% FBS, 1 mM sodium pyruvate, 2 mM GLUTamax, 1× nonessential amino acids, 0.1 mM 2-β-mercaptoethanol, 10 mM HEPES, 1% penicillin/streptomycin) + 1× Cell Stimulation Cocktail with Protein Inhibitors (eBioscience) for 3 h at 37 °C before staining. Nonviable cells were stained using Live/Dead Fixable Aqua. Cells were permeabilized with CytoFix/CytoPerm (BD), followed by intracellular staining in PermBuffer (eBioscience).

All antibodies were purchased from eBioscience unless otherwise indicated. Antibodies used were CD45-SB600, TCRb-APC-Cy7, MHCII-FITC, CD4-PE-Cy7, CD8a-AF700, CD8b-PE, IFNg-PerCP-Cy5.5, TNFa-APC, IL-17A-bv421 (BioLegend), CD3e-FITC, TCRg-FITC, B220-FITC, MHCII-APC-e780, CD11b-PerCP-Cy5.5, CD11c-AF700, CD64-APC (BioLegend), SiglecF-PE (BD), Ly6G-Pe-Cy7 (BD) and F4/80-e450. Sample data were acquired with an Attune NxT flow cytometer coupled with an Attune CytKick Max autosampler. Data were analyzed using FlowJo v.10. Antibody details and concentrations are included in Supplementary Table 1.

### Bacterial species characterization

While genomes are publicly available for the species described in this paper, and are represented by genomic species clusters in the Genome Taxonomy Database: A14 = *Anaerostipes sp000508985* (GCF_000508985.1: 98.76% ANI), A43 = *CAG-485 sp002362485* (GCF_003833075.1: 98.66% ANI), A60 = *Duncaniella sp001689575* (GCF_003762875.1: 96.19% ANI), A61 = *Alistipes sp002362235* (GCA_002362235.1: 98.54% ANI)). These species have not yet been characterized, nor have these names been validly published according to the International Code of Nomenclature of Prokaryotes. Because these species are uncharacterized, they are referred to as previously undescribed in this manuscript. Phenotypic data for the capacity of each species to utilize 95 substrates as sole carbon sources were attained using the BioLog platform (see BioLog analysis for a description of methodology). Isolated genomes were functionally annotated using eggNOG-mapper v.2 and InterProScan v.5. We describe these species and propose new names below.

#### Description of ‘*Alistipes okayasuensis*’ sp. nov

*Alistipes okayasuensis* (o’ka.ya.su.en.sis. N.L. masc./fem. adj. *okayasuensis*) is named after Isao Okayasu, who first described DSS colitis as a model for ulcerative colitis in mice. Phylogenomic analyses place strain A61^T^ in the *Alistipes* cluster. The digital DNA–DNA hybridization (dDDH) value between *A. onderdonkii* and *A. putredinis* is 24.4%, while dDDH values between strain A61^T^, *A. onderdonkii* and *A. putredinis* are 25.2 and 21.4%, respectively. The G + C content difference between the genome of strain A61^T^ and *A. onderdonkii*, its closest phylogenomic neighbor, is 1.39%, while their 16S rRNA gene sequence identity is 96.03%. These analyses strongly indicate that ‘*Alistipes okayasuensis*’ is a separate species within the genus *Alistipes*.

This strain is a strict anaerobe. Carbon source utilization analysis was combined with reconstruction of Kyoto Encyclopedia of Genes and Genomes (KEGG) metabolic pathways (55.58% of 2,022 predicted open reading frames (ORFs) could be annotated) to functionally characterize the strain. The strain encodes 47 genes predicted to be carbohydrate-active enzymes (CAZymes), including 29 glycoside hydrolases: seven beta-glucosidases (GH3, EC 3.2.1.21); five beta-galactosidases (EC 3.2.1.23); five endohydrolytic alpha-glucosidases (GH13), including four alpha-amylases (EC 3.2.1.1); three exohydrolytic alpha-glucosidases (GH31, EC 3.2.1.20); three cellulases (EC 3.2.1.4); three mannan endo-1,4-beta-mannosidases (EC 3.2.1.78); and an alpha-l-fucosidase (EC 3.2.1.51). Despite this predicted hydrolytic potential, the strain shows limited capacity to utilize disaccharides and oligosaccharides as sole carbon sources. While the strain tested positive for utilization of alpha-d-lactose, sucrose and maltotriose, it tested negative for utilization of d-cellobiose, dextrin, cyclodextrin, gentiobiose, lactulose, maltose, d-melezitose, d-melibiose, palatinose, d-raffinose, stachyose, d-trehalose, fucose or turanose.

The strain is predicted to encode enzymes for metabolism of l-histidine to l-glutamate via 4-Imidazolone-5-phsophate (EC 4.3.1.3, EC 4.2.1.49, EC 3.5.2.7, EC 2.1.2.5). In support of these predictions, the strain is capable of utilizing urocanate as a sole carbon source. In keeping with other members of the genus *Alistipes*, this strain is predicted to encode a putative tryptophanase (EC 4.1.99.1) that would allow it to hydrolyze tryptophan to indole. The strain is also predicted to encode a butyrate phosphotransferase (EC 2.3.1.19) and a butyrate kinase (EC 2.7.2.7), indicating its potential to metabolize butyrate. The genome is 2,329,524 bp with a G + C content of 56.45 mol%. We propose the name ‘*Alistipes okayasuensis*’ for this previously undescribed species, in reference to the origins of the DSS colitis model and reflecting this species’ association with poor outcomes of this disease model. The type strain is A61^T^ (=CCUG 75087^T^ =SM 112987^T^).

#### Description of ‘*Anaerostipes faecis*’ sp. nov

*Anaerostipes faecis* (fae’cis. L. gen. fem. n. *faecis*, of feces, referring to fecal origin). The closest phylogenomic neighbor of strain A14^T^ is *Anaerostipes caccae*, with a dDDH value of 48.40%. The pairwise 16 S rRNA gene sequence identity of A14^T^ and *A. caccae* DSM 14662^T^ is 98.16%. These analyses indicate that A14^T^ is a separate species within the genus *Anaerostipes*. The genome is 3,200,246 bp with a G + C content of 43.77 mol%. We propose the name ‘*Anaerostipes faecis*’ for this new species. The type strain is A14^T^ (=CCUG 75084^T^).

The strain is strictly anaerobic. Reconstruction of KEGG metabolic pathways (55.4% of 3,222 predicted ORFs could be annotated) indicates that the strain can produce butyrate via a butyrate phosphotransferase/butyrate kinase operon (EC 2.7.2.7, EC 2.3.1.91), in keeping with the genus type species *A. caccae*. Likewise, the strain is predicted to encode an arginine dihydrolase (EC 3.5.3.6). In contrast to the genus description, however, this strain is predicted to encode neither an alpha-galactosidase (EC 3.2.1.22) nor a phosphoamidase (EC 3.9.1.1).

The strain shows limited capacity to utilize monosaccharides as sole carbon sources: it had a robust phenotype for utilization of 3-methyl-d-glucose but tested negative for its ability to utilize other monosaccharides including d-fructose, d-galactose, d-mannose or d-glucose as sole carbon sources. Notably, the strain is predicted to encode the complete Leloir pathway for galactose metabolism, as well as multiple beta-galactosidases (EC 3.2.1.23), indicating that it might lack a galactose transporter but could metabolize galactose in different contexts such as downstream of other pathways of carbohydrate metabolism. The strain can utilize some disaccharides, testing positive for sucrose utilization, potentially via multiple oligo-1,6-glucosidases (EC 3.2.1.10), d-trehalose utilization, potentially via three trehalases (EC 3.2.1.28, GH37, GH65) and turanose utilization. However, the strain tested negative for its ability to utilize alpha-d-lactose, lactulose, maltose, d-melibiose, gentiobiose, d-cellobiose or palatinose. Additionally, the strain tested negative for utilization of the trisccharides maltotriose, d-melezitose and d-raffinose and the tetrasaccharide stachyose—potentially due to the absence of a predicted alpha-galactosidase. The strain could utilize beta-cyclodextrin, but tested negative for utilization of dextrin and alpha-cyclodextrin. The strain is capable of utilizing alpha-ketovaleric acid and d-malic acid, as well as certain amino acids and derivatives including l-threonine, l-asparagine, l-serine and the dipeptides glycyl-l-proline and l-alanyl-l-threonine. The strain tested negative for utilization of l-glutamine, l-glutamate, l-valine and l-alanine.

#### Description of ‘*Duncaniella muricolitica*’ sp. nov

*Duncaniella muricolitica* (mu.ri.co’li.ti.ca. L. gen. n. *muris*, of the mouse; Gr. n. *kolon*, colon; Gr. suff. *-tikos -ê -on*, suffix used with the sense of pertaining to; N.L. fem. adj. *muricolitica*, pertaining to the colon of the mouse). The closest phylogenetic neighbor, according to pairwise 16 S rRNA gene analyses, is *Duncaniella dubosii* with a sequence identity of 94.04%. The 16 S gene sequence identity values between *D. dubosii* H5^T^ and other species of the genus, *D. muris* DSM 103720^T^ and *D. freteri* TLL-A3^T^, are 95.14 and 93.00%, respectively, indicating that A60^T^ is a separate species within the *Duncaniella* cluster. The dDDH value between A60^T^ and *D. dubosii* H5^T^ is 28.10%, confirming a separate species status.

Recent work benchmarking an average nucleotide identity^1^ approach to define genus boundaries found that the genus inflection points of members of the order Bacteroidales had relatively low values for alignment fraction (AF) and ANI (AF/ANI: *Bacteroides fragilis*, 0.215/71.23; *Porphyromonas asaccharolytica*, 0.105/68.12; *Prevotella melaninogenica*, 0.195/72.07). The corresponding values between A60^T^ and the genus type species *D. muris* are 0.243/80.85, providing further evidence that A60^T^ belongs to the genus *Duncaniella*.

The strain is an obligate anaerobe. Reconstruction of KEGG metabolic pathways (47.2% of 3,461 predicted ORFs could be annotated) indicated that strain is enriched for enzymes with glycoside hydrolase activity, encoding 47 in total. The strain is able to utilize dextrin, beta-cyclodextrin and multiple oligosaccharides including d-melezitose, maltotriose, amygdalin, maltose, gentiobiose (EC: 3.2.1.21), turanose and d-cellobiose (EC: 2.4.1.20; EC: 3.2.1.21). The strain is capable of utilizing d-raffinose, and is predicted from its genome to produce sucrose as an intermediate (EC: 3.2.1.22, EC: 3.2.1.20) rather than stachyose or melibiose. Additionally, the strain tested negative for utilization of both stachyose and melibiose. In our study the strain tested negative for an ability to utilize lactose as a single carbon source, even though it encodes seven predicted beta-galactosidases (EC 3.2.1.23). The strain is predicted to encode 36 glycosyltransferase enzymes, among which are seven GT2 family enzymes including two dolichyl-phosphate beta-d-mannosyltransferases (EC: 2.4.1.82).

The strain can utilize a variety of monosaccharides as single carbon sources, including alpha-d-glucose, alpha-d-glucose 1-phosphate, 3-methyl-d-glucose, d-galactose, alpha-methyl-d-galactose, d-mannose, adonitol, arabinose, d-gluconate, d-arabitol and d-sorbitol. It tested negative for an ability to utilize fucose, alpha-d-lactose, d-fructose, lactate, lactulose, d-trehalose or l-rhamnose as sole carbon sources. The strain is predicted to encode the pathway for metabolism of l-histidine to l-glutamate via 4-Imidazolone-5-phsophate (EC 4.3.1.3, EC 4.2.1.49, EC 3.5.2.7, EC 2.1.2.5), and tested positive for utilization of urocanate. The genome is 4,055,027 bp with a G + C content of 50.9 mol%. Following functional and genomic characterization of our isolate, we propose the name ‘*Duncaniella muricolitica*’ for this species in reference to its association with poor prognosis in the murine DSS model of colitis. The type strain is A60^T^ (=CCUG 75086^T^ =DSM 112986^T^).

#### Description of ‘*Sangeribacter*’ gen. nov

*Sangeribacter* (San.ge.ri.bac’ter. N.L. gen. masc. n. *Sangeri*, of Sanger; N.L. masc. n. *bacter*, rod; N.L. masc. n. *Sangeribacter*, a rod-shaped bacterium named after Frederick Sanger (1918–2013) and the institute where this genus was first described). 16 S rRNA gene sequence analyses place this strain in the Muribaculaceae family, and ‘*Sangeribacter*’ possesses all the features of this family. The pairwise sequence identity of the 16 S rRNA gene of A43^T^ with 16 S genes from *Paramuribaculum intestinale* DSM 100749^T^, *Muribaculum intestinale* YL27^T^ and *Duncaniella muris* DSM 103720^T^ is 88.90, 88.22 and 87.39%, respectively. The dDDH value between A43^T^ and *Paramuribaculum intestinale* DSM 100749^T^ is 35.5% and the difference in G + C mol% is 6.37%. Together these analyses strongly indicate that ‘*Sangeribacter*’ is a separate genus. The type species, *Sangeribacter muris*, is one of the most dominant species in the mouse gut microbiota, representing up to 60% of classified reads in some mouse fecal shotgun metagenome samples. It is also highly prevalent, present in 77% of 1,926 samples.

#### Description of ‘*Sangeribacter muris*’ sp. nov

*Sangeribacter muris* (mu’ris. L. gen. masc./fem. n. *muris*, of the mouse, the species was first isolated from a mouse). The strain is a strict anaerobe. It is predicted to encode 66 CAZymes including 42 glycoside hydrolases. The strain tested positive for utilization of a wide repertoire of glycans and carbohydrates, including dextrin (EC 3.2.1.3), alpha- and beta-cyclodextrin, stachyose (EC 3.2.1.22), d-raffinose (EC 3.2.1.26), maltotriose, d-melezitose, amygdalin (EC 3.2.1.22), d-cellobiose (EC 3.2.1.21, EC 2.4.1.20), gentiobiose (EC 3.2.1.21), d-melibiose, palatinose, d-trehalose (EC 2.7.1.201, EC 2.4.1.64, EC 3.1.3.12), turanose, sucrose (EC 2.7.1.211), d-glucosaminate, lactulose, alpha-d-lactose, alpha-d-glucose, alpha-D-glucose 1-phosphate, 3-methyl-d-glucose, d-glucosaminic acid, alpha- and beta-methyl-d-glucoside, d-fructose (EC 2.7.1.4), d-mannitol (EC 1.1.1.67), d-mannose, d-galactose, alpha- and beta-methyl-d-galactoside, d-galacturonic acid, l-sorbitol (EC 1.1.1.14) and l-fucose (EC 3.2.1.51). This strain tested negative for its ability to utilize adonitol, dulcitol or d-gluconate as single carbon sources.

The strain tested positive for its ability to utilize certain amino acids and their derivatives, including l-phenylalanine, l-asparagine, l-glutamate, l-glutamine, l-alanine and l-valine, as well as dipeptides including l-alanyl-l-threonine, l-alanyl-l-glutamine, l-alanyl-l-histidine, glycyl-l-aspartate, glycyl-l-glutamine and glycyl-l-proline. It tested negative for an ability to utilize glycyl-l-proline, l-serine, l-aspartate or l-threonine as single carbon sources. The strain is predicted to encode a tryptophanase (EC 4.1.99.1), indicating that it has potential to hydrolyze tryptophan to indole. The strain tested negative for utilization of propionate or hydroxybutyrate as sole carbon sources, indicating that it may not consume these short-chain fatty acids in vivo. The genome is 3,532,505 bp with a G + C content of 46.70 mol%. We propose the name ‘*Sangeribacter muris*’ for this new species, reflecting its high abundance and prevalence in mice and its initial isolation from a murine host. The type strain is A43^T^ (=CCUG 75085^T^)

### BioLog analysis

Isolates were streaked on YCFA agar media and grown overnight. Cotton swabs were used to remove colonies that were then inoculated into AN-IF Inoculating Fluid (Technopath, no. 72007) to a turbidity of 65% using a turbidimeter. Then, 100 µl was pipetted into each well of Anaerobe AN Microplates (Technopath, no. 1007) containing 95 different carbon sources. The plates were sealed in PM Gas Bags (Technopath, no. 3032) and run on the Omnilog system for 24 h. For each isolate, between three and five replicates were run on different days from different starting colonies. Data were analyzed using the CarboLogR application^34^. The quality-filtered CarboLogR output is provided in Supplementary Table 2.

### Reporting Summary

Further information on research design is available in the Nature Research Reporting Summary linked to this article.

## Supplementary information

Reporting Summary

Supplementary Tables Supplementary Table 1: Antibody dilutions for flow cytometric analysis. Supplementary Table 2: Carbon source utilization. Numbers (0 or 1) indicate the ability of isolates to utilize single carbon sources based on a minimum of three repeats using the Biolog AN Microplate

Source Data Fig. 2 Statistical Source data.

Source Data Fig. 3 Statistical Source data.

## Data Availability

Metagenomic sequencing data were deposited in ENA under project no. PRJEB50449. Key strains were deposited in the University of Gothenburg Culture Collection (CCUG) or the DSMZ German Collection of Microorganisms (DSM) under the following identifiers: *Alistipes okayasuensis* (CCUG 75087^T^/DSM 112987^T^), *Duncaniella muricolitica* (CCUG 75086^T^/DSM 112986^T^), *Sangeribacter muris* (CCUG 75085^T^) and *Anaerostipes faecis* (CCUG 75084T). Source data are provided with this paper.

## Code availability

No custom code was used in this research.
