# Supplementary material for: Identification of gut microbial species linked with disease variability in a widely used mouse model of colitis
Source: Nat Microbiol. 2022 Apr 1;7(4):590–9. doi: 10.1038/s41564-022-01094-z (PMC8975739; doi:10.1038/s41564-022-01094-z)
Supplement: Supplementary file 1 — Reporting Summary [file 41564_2022_1094_MOESM1_ESM.pdf]

nature research

Corresponding author(s): Trevor Lawley
Last updated by author(s): 23_02_2022

Reporting Summary

Nature Research wishes to improve the reproducibility of the work that we publish. This form provides structure for consistency and transparency in reporting. For further information on Nature Research policies, see our Editorial Policies and the Editorial Policy Checklist.

Statistics

For all statistical analyses, confirm that the following items are present in the figure legend, table legend, main text, or Methods section.

n/a	Confirmed	
☐	☒	The exact sample size (*n*) for each experimental group/condition, given as a discrete number and unit of measurement
☒	☐	A statement on whether measurements were taken from distinct samples or whether the same sample was measured repeatedly
☐	☒	The statistical test(s) used AND whether they are one- or two-sided<br>*Only common tests should be described solely by name; describe more complex techniques in the Methods section.*
☐	☒	A description of all covariates tested
☐	☒	A description of any assumptions or corrections, such as tests of normality and adjustment for multiple comparisons
☐	☒	A full description of the statistical parameters including central tendency (e.g. means) or other basic estimates (e.g. regression coefficient) AND variation (e.g. standard deviation) or associated estimates of uncertainty (e.g. confidence intervals)
☒	☐	For null hypothesis testing, the test statistic (e.g. *F*, *t*, *r*) with confidence intervals, effect sizes, degrees of freedom and *P* value noted<br>*Give P values as exact values whenever suitable.*
☒	☐	For Bayesian analysis, information on the choice of priors and Markov chain Monte Carlo settings
☒	☐	For hierarchical and complex designs, identification of the appropriate level for tests and full reporting of outcomes
☒	☐	Estimates of effect sizes (e.g. Cohen's *d*, Pearson's *r*), indicating how they were calculated

Our web collection on statistics for biologists contains articles on many of the points above.

Software and code

Policy information about availability of computer code

Data collection	*Provide a description of all commercial, open source and custom code used to collect the data in this study, specifying the version used OR state that no software was used.*
Data analysis	Software tools used for data analysis were as follows. Kraken2/Bracken, vegan v2.5-6 package, ggpubr package, mothur MiSeq SOP v.1.42.3, SILVA v.13217 and FastTree v.2.1.10

For manuscripts utilizing custom algorithms or software that are central to the research but not yet described in published literature, software must be made available to editors and reviewers. We strongly encourage code deposition in a community repository (e.g. GitHub). See the Nature Research guidelines for submitting code & software for further information.

Data

Policy information about availability of data

All manuscripts must include a data availability statement. This statement should provide the following information, where applicable:

- Accession codes, unique identifiers, or web links for publicly available datasets
- A list of figures that have associated raw data
- A description of any restrictions on data availability

All sequencing data are being deposited with the ENA, and identifiers will be included prior to publication.

Field-specific reporting

Please select the one below that is the best fit for your research. If you are not sure, read the appropriate sections before making your selection.

☒ Life sciences ☐ Behavioural & social sciences ☐ Ecological, evolutionary & environmental sciences

For a reference copy of the document with all sections, see nature.com/documents/nr-reporting-summary-flat.pdf

Life sciences study design

All studies must disclose on these points even when the disclosure is negative.

Sample size	579 genetically identical mice
Data exclusions	No data was excluded
Replication	All attempts at replication were successful with replicate numbers described for each experiment performed.
Randomization	Not relevant to this study design
Blinding	Not relevant to this study design

Reporting for specific materials, systems and methods

We require information from authors about some types of materials, experimental systems and methods used in many studies. Here, indicate whether each material, system or method listed is relevant to your study. If you are not sure if a list item applies to your research, read the appropriate section before selecting a response.

Materials & experimental systems

n/a	Involved in the study	
☐	☒	Antibodies
☒	☐	Eukaryotic cell lines
☒	☐	Palaeontology and archaeology
☐	☒	Animals and other organisms
☒	☐	Human research participants
☒	☐	Clinical data
☒	☐	Dual use research of concern

Methods

n/a	Involved in the study	
☒	☐	ChIP-seq
☐	☒	Flow cytometry
☒	☐	MRI-based neuroimaging

Antibodies

Antibodies used	All antibodies were purchased from eBioscience/Invitrogen/Thermo unless otherwise indicated. Antibodies used were CD45-SB600, TCRb-APC-Cy7, MHCII-FITC, CD4-PE-Cy7, CD8a-AF700, CD8b-PE, IFNg-PerCP-Cy5.5, TNFa-APC, IL-17A-bv421 (BioLegend), CD3e-FITC, TCRg-FITC, B220-FITC, MHCII-APC-e780, CD11b-PerCP-Cy5.5, CD11c-AF700, CD64-APC (BioLegend), SiglecF-PE (BD), Ly6G-Pe-Cy7 (BD) and F4/80-e450.
Validation	All antibodies were validated by their respective manufacturer.

Animals and other organisms

Policy information about studies involving animals; ARRIVE guidelines recommended for reporting animal research

Laboratory animals	SPF wild type C57BL/6N mice; 297 male, 282 female; median age 11 weeks; SD 12 days.
Wild animals	The study did not involve wild animals
Field-collected samples	The study did not involve field-collected samples
Ethics oversight	Mice were maintained under specific pathogen-free conditions at a Home Office-approved facility with all procedures carried out in accordance with the United Kingdom Animals (Scientific Procedures) Act of 1986.

Note that full information on the approval of the study protocol must also be provided in the manuscript.

Flow Cytometry

Plots

Confirm that:

☒ The axis labels state the marker and fluorochrome used (e.g. CD4-FITC).

☒ The axis scales are clearly visible. Include numbers along axes only for bottom left plot of group (a 'group' is an analysis of identical markers).

☒ All plots are contour plots with outliers or pseudocolor plots.

☒ A numerical value for number of cells or percentage (with statistics) is provided.

Methodology

Sample preparation	The large intestines were opened longitudinally, cleared of faeces, and then washed three times in cold PBS. To isolate the intraepithelial immune populations, the large intestines were cut into 1cm pieces, washed in cold PBS, and then incubated in 10 mL of PBS + 1 mM dithiothreitol for 10 minutes. Tissues were manually disrupted via shaking and then strained, collecting the supernatant. Tissues were then incubated in 10 ml of PBS + 30 mM EDTA + 10 mL HEPES at 37°C at 200 rpm, before being shaken and strained. The supernatants from these two steps were pooled, filtered at 70 µm and the filtrate fractionated using a discontinuous Percoll gradient (80%/40%). Epithelial cells were isolated from the surface of the Percoll, and the intraepithelial immune cells isolated from the interface.<br><br>To access the lamina propria compartment, the tissues were manually chopped and digested in HBSS + 25 mM HEPES + 1 mM sodium pyruvate containing 0.05 mg/mL Collagenase VIII (Sigma) and 50 µg/ml DNase I (Sigma) for 1 hour at 37°C at 80 rpm. Samples were mechanically disrupted and filtered at 70 µm. The filtrate was fractionated using a discontinuous Percoll gradient (80%/40%). Lamina propria immune cells were isolated from the interface.
Instrument	Attune NxT flow cytometer coupled with an Attune CytKick Max autosampler.
Software	Data were analysed using FlowJo v10.
Cell population abundance	Frequency of relevant populations shown on the plots.
Gating strategy	Viable hematopoietic cells were gated as singlets from FSC/SSC and then gated as CD45+, viability dye-. Further cell subtypes were gated on type-specific markers as indicated in the figure legends.

☐ Tick this box to confirm that a figure exemplifying the gating strategy is provided in the Supplementary Information.
